# Retraction: Limited immune surveillance in lymphoid tissue by cytolytic CD4+ T cells during health and HIV disease

**DOI:** 10.1371/journal.ppat.1013442

**Published:** 2025-08-27

**Authors:** The PLOS Pathogens Editors

The *PLOS *Pathogens** Editors retract this article [1] due to concerns about compromised peer review and undisclosed competing interests between the Academic Editor and the authors. These concerns call into question the reliability and validity of the reported results. We regret that the issues were not identified prior to the article’s publication.

MB, DPP, KD, JKS, MAE, MJ, EJW, ACK, and MRB did not agree with the retraction. SN, LMM, MS, NA, PMDRE, YAT, KN, MAR, HS, PN, AS, DHC, AN, MLR, SGD, GRT, and YS either did not respond directly or could not be reached.

In addition to the issues above, concerns were raised regarding the informed consent and registration of the protocol used for collection of clinical samples at INER-CIENI, Mexico. During editorial follow-up, copies of the informed consent, ethical approval, and registration documents with the Mexican authority COFEPRIS were provided. In light of the peer review and competing interest concerns with this article, PLOS did not investigate the informed consent and registration concerns further.
